# Documenting plans for care: advance care directives and the 7-step pathway in the acute care context

**DOI:** 10.1186/s12904-021-00838-8

**Published:** 2021-09-09

**Authors:** Gregory Brian Crawford, Katherine Hodgetts, Teresa Burgess, Jaklin Eliott

**Affiliations:** 1 Present Address: Northern Adelaide Local Health Network, C/- Modbury Hospital, Smart Road, Modbury, 5092 Australia; 2 Discipline of Medicine, University of Adelaide, Adelaide, 5005 Australia; 3 School of Public Health, University of Adelaide, Adelaide, 5005 Australia

**Keywords:** Advance care planning, Advance care directives, Integrated care plans, Qualitative study

## Abstract

**Background:**

Advance care planning involves the discussion and documentation of an individual’s values and preferences to guide their future healthcare should they lose capacity to make or communicate treatment decisions. Advance care planning can involve the individual’s completion of an Advance Care Directive (ACD), a legislated and common-law instrument which may include appointment of a substitute decision-maker and binding refusals of treatment. In South Australia, ACDs intersect in the acute-care context with the Resuscitation Plan 7-Step Pathway (7-SP), an integrated care plan written for and by clinicians, designed to organise and improve patients’ end-of-life care through the use of structured documentation. Here, we examine the perspectives of healthcare professionals (HCPs) within a hospital setting on the practical integration of ACDs and the 7-SP, exploring the perceived role, function, and value of each as they intersect to guide end-of-life care in an Australian hospital setting.

**Methods:**

Qualitative data were collected via eight focus groups with a total of 74 HCPs (acute care, and oncology specialists; medical intern; general and emergency nurses; social workers) across two hospitals. Audio recordings were transcribed and thematically analysed.

**Results:**

HCPs viewed ACDs as a potentially valuable means of promoting patient autonomy, but as rarely completed and poorly integrated into hospital systems. Conversely, the process and documentation of the 7-SP was perceived as providing clarity about clinicians’ responsibilities, and as a well-understood, integrated resource. Participants sometimes exhibited uncertainty around which document takes precedence if both were present. Sometimes, the routinisation of the 7-SP meant it was understood as the ‘only way’ to determine patient wishes and provide optimal end-of-life care. When this occurs, the perceived authority of ACDs, or of patients’ choice not to participate in end-of-life discussions, may be undermined.

**Conclusions:**

The intersection of ACDs and the 7-SP appears problematic within acute care. Clinicians’ uncertainty as to whether an ACD or 7-SP takes precedence, and when it should do so, suggests a need for further clarity and training on the roles of these documents in guiding clinical practice, the legislative context within which specific documentation is embedded, and the dynamics associated with collaborative decision-making in end-of-life care.

## Introduction

In Australia, advance care planning has been increasingly advocated as a means of improving end-of-life care through the promotion of patient autonomy [1–3]. Advance care planning is argued to support best-practice patient-centred care, enhancing quality of life for patients and families [4, 5] while reducing the personal and economic costs of unwanted and futile interventions as patients approach the end of life [6, 7].

In general terms, advance care planning involves the discussion and documentation of an individual’s values and preferences to guide their future healthcare should they lose the capacity to make or communicate treatment decisions [8, 9]. This can involve the individual’s completion of an Advance Care Directive (ACD), a legislated and common-law instrument which may include the appointment of a substitute decision-maker and binding refusals of treatment [9]. It may also involve the articulation of personal values, desires and more general end-of-life care preferences designed to guide health decision-making in the event of future incapacity [2].

In South Australia, ACDs intersect in the acute care context with the Resuscitation Plan 7-Step Pathway (7-SP). Underpinned by the Resuscitation Planning Policy Framework with which compliance is explicitly framed as “mandatory” ( [10] p.1), the 7-SP is an example of an integrated care plan, designed to organise and improve patients’ end-of life-care through the use of structured documentation accessible by relevant clinicians across healthcare settings [11]. The 7-SP stipulates that if an in-patient meets any of five specified clinical triggers, medical professionals should consult with them, or their nominated substitute decision-maker/s, to create and document a clinical plan for their end-of-life care. Documentation of the 7-SP encourages recommendations or refusals of treatment in accordance with medical opinion and the patient’s wishes (which may or may not be documented in an existing ACD). This “standardised process for screening, developing and implementing” end-of-life care plans ( [12] p. 3) culminates in the completion of a 7-SP Alert Form—a hard copy document with tickboxes through which a treating doctor may communicate any limits of care (including not for CPR, intubation, or admission to an Intensive Care Unit) and stipulate that the treatment plan is valid “for the current admission only” or “indefinitely until revoked” ( [12], p. 9).

Since the introduction of the 7-SP, research involving a case-note audit of a South Australian hospital site has indicated that its use has been associated with increased rates of documented discussions with patients/substitute decision-makers, recorded limitations to care, and patients identified as not for CPR. Indeed, Dignam et al. have argued that, since its introduction, the 7-SP “has improved patient autonomy by respecting patients’ wishes and providing greater clarity about treatment decisions” ( [13] p. 28). However, a subsequent mixed-methods study within a South Australian hospital reported that ambiguity in the terminology used in 7-SP documentation is likely to undermine attempts to ensure that patients’ preferences are accurately captured and upheld [14]. Further, it suggested that level of seniority of the completing clinician influenced both the perceived purpose of the resuscitation plan, and the level of confidence other clinicians might place in the resulting documentation [14].

As outlined in the Policy Framework, where a patient has completed an ACD and meets clinical criteria to trigger documentation of a 7-SP, these documents should, in combination, “translate” into a Clinical Care Plan that relevant clinicians can action as necessary during the patient’s admission ( [12] p. 9). However, how this works in practice has not yet been examined. In this paper, we will analyse the perspectives of healthcare professionals (HCPs) within a hospital setting on the relative role, value, and function of ACDs versus the 7-SP in meeting the goals of advance care planning, including issues of autonomy, clarity and transparency in end-of-life care. In addition, we will attend to the practical integration of ACDs and the 7-SP in situ: how the ‘translation into action’ of these documents is practically negotiated in the context of acute care.

## Method

### Design and approvals

This study provides a thematic analysis of focus groups with HCPs around the role, implementation and merits of ACDs and the 7-SP in the context of acute care. Focus group data were collected as part of a broader audit of advance care planning policy and practice in two South Australian hospitals. The hospital sites selected are State-government operated, serving culturally and ethnically diverse populations in outer-metropolitan Adelaide.

### Ethics approval, recruitment and consent to participate

This study complied with the Declaration of Helsinki and was approved by the South Australian Department for Health and Wellbeing Human Research Ethics Committee (DHW HREC, Approval reference: HREC/17/SAH/128). To minimise interference with the hospitals’ clinical operation, the project researchers met with the Clinical Governance Committee, Director of Nursing, Medical Intern Placement Coordinators, and other relevant managers to seek approval and identify suitable times for the focus groups. Flyers were then circulated throughout the hospitals to invite HCPs to contact authors 1 & 2 if they were willing to discuss issues associated with advance care planning and ACDs. All HCPs onsite were eligible to participate. Signed informed consent was obtained from all study participants in writing before the commencement of each group. Participants were informed that they were free to withdraw from the study at any time and that anonymity and confidentiality would be maintained through the use of pseudonyms.

### Focus groups

Data were collected via focus groups, an approach that facilitates group interaction, providing opportunity for participants to explore and clarify their own and others’ perspectives [15]. Focus groups were conducted between January and July 2019 and comprised semi-structured discussions guided by questions arising from a literature review and the findings of a case-note audit of advance care planning documentation previously conducted at each site. Discussions lasted 60-90 min, and were facilitated by authors 1 & 2 in private, on-site rooms away from the clinical workplace at times convenient to participants (typically lunchtimes or after standard work hours; focus groups with nurses were scheduled within a regular program of education sessions). Focus groups were digitally recorded and transcribed verbatim, and data de-identified before analysis to protect confidentiality.

### Participants

Eight focus groups were conducted with a total of 74 participants (see Table 1). Focus groups were discipline-specific to increase group homogeneity and avoid power imbalances that could cause participant self-censorship or reticence to share [16]. Maximum variation sampling [17] was chosen to elicit diverse perspectives across a range of health professional with direct experience of advance care planning in acute care. While most participants worked across both the selected hospital sites, focus groups were primarily held at Hospital 1 for reasons of scheduling and available space.

**Table 1:** Composition of focus groups

	Focus group no.	Focus group members	No. of participants
**Hospital 1**	1	Nurses: Emergency	9
	2	Nurses: General	25
	3	ICU specialists	5
	4	Oncologists	4
	5	Social workers	7
	6	Interns/RMOs	1
**Hospital 2**	7	Nurses: General	17
	8	Social workers	6

### Data analysis

Focus group transcripts were analysed thematically [18, 19], underpinned by a critical realist framework [20, 21]. Analysis was grounded in the assumption that focus group outputs reflect both the interactional context in which they were generated, as well as broader patterns of sense making that can have ideological, organisational and material consequences in end-of-life care settings.

All transcripts were reviewed during a process of data familiarisation, and all participant talk about the relative roles, merits, and functions of ACDs and 7-SP documentation were extracted. Coding was then undertaken via an iterative process in which relevant extracts were read and re-read before recurrent patterns were defined. Codes were then collapsed into like categories constituting recurrent themes. Coding and initial theme development was undertaken by author 2 and refined through discussion and re-reading of a sub-set of transcripts with authors 2 & 3. Themes identified were subsequently analysed with regard to their specific content and broader implications for advance care planning practice and policy. Quotes presented are chosen as illustrative, concise examples of participant perspectives.

## Results

Two key themes and associated sub-themes were identified (see Table 2 below). The first theme encompassed participants’ views on the relative value of ACDs and the 7-SP in meeting the goals of advance care planning. The second centred on practical concerns about the integration of these documents into clinical practice in acute care contexts.

**Table 2:** Summary of themes and sub-themes

Key Theme	Sub-theme
**(Relative) Value: ACD vs 7-SP**	‘Ongoing preferences’ vs ‘informed decisions’
	‘Static’ vs ‘dynamic’ documentation
**Complexity of integration into practice**	Consequences of ‘routine’ provision
	Enhancing ‘clarity’
	Enhancing ‘transparency’

### (Relative) value: advance care directives vs the 7-step pathway

Across the dataset, HCPs consistently conflated ACDs and 7-SP documentation, conceptualising both as examples of an ‘advance care plan’. There were, however, discernible differences in their views on the value and role of each document. For example, ACDs were primarily valued as promoting patient autonomy and supporting quality of care, whereas the 7-SP with Alert Form was understood to promote clear communication between treating HCPs.

Across disciplines, participants agreed in principle that ACDs constitute a valuable vehicle for patients to express healthcare and treatment preferences, and to support *“quality of care as well as patients’ rights and … dignity”* (ICU specialist). HCPs saw the main value of ACDs as providing a means of initiating and scaffolding end-of-life conversations that may be otherwise difficult to broach and to document, while symbolically introducing the possibility of death as an outcome.

[O]ne of the first questions I’ve seen some clinicians ask patients or families is do you have an advance care directive when they’re broaching the subject of what the resus sort of wishes are. And if they don’t know what that is or if you know, you know they haven’t had that done before then that’s probably a good sort of way of broaching the subject at least. I think then things click that you’re talking about [the] end of life. (Intern)

HCPs agreed that ACDs can serve to communicate a patient’s *“essential preferences”* (intern) to inform care and treatment choices at the end of life. The nomination of a substitute decision-maker, facilitating a structured, timely process for the incorporation of family perspectives into treatment decision-making when patient capacity is lost, was also a perceived benefit of ACD completion.

Even just having the name of the Substitute Decision-Maker written down, it saves time and sometimes … it means the patient’s wishes are respected when they need to be. (social worker)

Ultimately, HCPs saw ACDs a means of ensuring that end-of-life decisions are genuinely *“about the patient”* (social worker) rather than the wishes of family members or doctors whose inclination may be to keep *“push, push, pushing”* (nurse) unwanted, aggressive or invasive treatment options.

Despite these advantages, HCPs reported a range of drawbacks of ACDs, including the observation that they are often inaccessible when required to inform clinical decision-making. When ACDs are present, HCPs noted that they are often unfinished, which they attributed to the complexities involved in completing, witnessing, and enacting the documentation. HCPs reported that documentation was more likely to be complete for patients coming from residential aged care facilities, which for some participants raised ethical concerns around influence, coercion, and capacity. For a number of HCPs, the inclusion within ACDs of general values and preferences (*“they’ll include things like ‘I want to die in my own bed’”* – ICU specialist) rendered them *“irrelevant”* (emergency nurse) in the context of decision-making around specific care and treatment options in the acute care setting.

The 7-SP was specifically understood to be a well-known, accessible *“case note for clinicians”* (ICU specialist) that creates clear communication channels (nurse) between treating professionals. Participants indicated that the 7-SP orients to patient-centred practice, but also to resource efficiency and costs to both the system and individuals (*“it’s designed to minimise resources, and [so we] don’t do something stupid to the patient”* – oncologist).

A key drawback identified by HCPs, however, was that the 7-SP/Alert Form, could often involve *“reinventing the wheel”* (social worker) in that a new conversation and documentation may be required for each acute admission regardless of whether another has been recently completed, or if an ACD is already in place. HCPs indicated that this revisiting of difficult conversations can undermine rapport between patients and their current treating team.

Sometimes [patients will] be like, “I want everything,” and they start getting upset. I'm not going to be there at midnight in ED telling them, “No, we are not going to do CPR.” It’s just when the time comes we won't be doing that, and, you know, you ruin your rapport if you start doing that. (oncologist)

In some scenarios, HCPs indicated that re-opening conversations previously settled in an ACD, in order to complete the 7-SP, can render patients vulnerable to pressure from family or others who may seek to influence established plans.

Families become very angry because Mum or Dad has an Advance Care Directive, and then we ask them to sit down and discuss again so that we can fill in the 7-Step Pathway, and then also distressed patients when their, particularly, sons and daughters don’t agree with their decisions that they made and they almost … try and bully Mum or Dad to change it. (nurse)

To address these issues, HCPs suggested that planning processes should be revised to enable an explicit combining of 7-SP and ACD documentation, or to enable the former, by default, to be ‘valid until revoked.’ The recording of 7-SP and/or ACD documentation on an electronic health record (e.g. My Health Record) was also broadly supported as a solution for some concerns.

### ‘Ongoing preferences’ vs ‘informed decision-making’

A key contrast between HCPs’ understandings of the value of ACDs and the 7-SP was in terms of the tensions that could arise when seeking to ensure that decisions made in acute care reflect both a patient’s ‘ongoing preferences’ and meet the requirements of ‘informed consent.’ On one hand, decisions recorded in an ACD were understood to be ‘genuine’: completed when a patient is in good health, and full capacity, and without the immediate pressure, anxiety and potential “irrationality” (social worker) engendered by an acute admission. On the other hand, HCPs indicated that the completion of the 7-SP on hospital admission more fully supports ‘informed decision-making’, in that clinical decisions require an understanding of the ramifications of treatment in light of a patient’s current health status, which cannot be fully understood or predicted in advance.

It depends how specific you were [when completing the ACD] and how many contingencies you put in your first one. You know, “I want everything done for me however in the meantime I've been diagnosed with a life-limiting illness.” How could you have known? But we know when we do the 7-SP. (oncologist)

In this sense, some HCPs indicated that ACDs should serve to inform the completion of the 7-SP, giving a general indication of the patient’s broader values (with regard, for example, to dignity, preserving life at all cost, etc.), while the 7-SP incorporates these notions into a care plan relevant to the specific admission. In contrast, the very specificity of the 7-SP (which can be recorded for use ‘indefinitely until revoked,’ but is usually recorded to guide care during a single acute admission, rarely leaving the hospital setting despite the policy intention that it should) was seen as a limitation by some HCPs, who emphasised the need for a document that can continue to guide care in the community setting.

In the community, that 7-Step Pathway doesn’t exist and patients’ wishes ... do need to be documented in a more broad sense and particularly in the community where they have interaction with many health professionals and ambulance services and GPs and nurses …. there is a major gap. (oncologist)

### ‘Static’ vs ‘dynamic’ documentation

In line with the above concern was a dilemma arising when ACDs and 7-SP forms were respectively characterised as ‘static’ versus ‘dynamic’. Although clinicians indicated the importance of patients’ communicating long-standing and ongoing preferences in ACD as a means of ensuring autonomy, there was also concern that ACDs might be completed too far in advance. For example, without ongoing revision in light of changing circumstances, it was argued that ACDs could potentially be rendered *“useless”* (oncologist) as disease progresses and physical and mental conditions change along an illness trajectory.

For example, HCPs indicated that a one-time completion of an ACD document may not take into account issues around whether patients can provide or withhold consent for future treatments they are unaware may become relevant to their condition, the ramifications of which may not be possible to understand in the early stages of illness. Similarly, HCPs suggested that ‘static’ ACDs cannot take into account how people will feel about their future treatment options if their perspective alters as their disease progresses or general health state deteriorates. By contrast, the 7-SP was viewed as potentially more dynamic and responsive to changes in the patient’s condition and prognosis.

The 7-SPs [are] dynamic. They can change. Do you want to put more in? Do you want to take some out? In the 7-SP you can. Did your circumstances change? And so, the risk … of having an ACD is you end up with these redundant, worthless documents where … your preferences around end-of-life are not what you want. (oncologist)

### Complexity of integration into clinical practice

The thorough integration of the 7-SP and Alert Form into hospital clinical practice was argued to be its strongest advantage over ACDs. HCPs reported that the integration of the 7-SP has arisen from systemic support, including the allocation of resources to education and training, which has seen resultant clarity among HCPs regarding their roles and responsibilities in the completion of documentation and implementation of recorded plans. Although the 7-SP Policy Directive outlines “a role for all members of the health care team” ( [10] p. 8) HCPs reported that 7-SP documentation is routinely completed by clinicians (*“usually the senior doctor”* – nurse) as a matter of priority within 24 h of the acute admission. Across disciplines, HCPs reported widespread understanding of physical and electronic systems through which the 7-SP Alert Form is completed, stored, accessed, and supported by an electronic system of alerts. Despite concerns about the accessibility of 7-SP documents between hospitals and across separate admissions, the 7-SP was generally understood to be vastly more accessible than the ACD, which HCPs indicated is rarely completed, difficult to access when needed (being regularly filed with a lawyer rather than a relevant health practitioner) and often confused by patients with their will or financial documents.

Notably, across the dataset, there were instances wherein HCPs demonstrated confusion around the legal standing of 7-SP Alert Form and ACDs, identifying areas of particular complexity when both documents were present, complete and clinically relevant. For example, some HCPs argued for the prioritisation of the 7-SP over an ACD on both clinical or procedural grounds *(“It’s informed by a member of the current treating team”* – ICU specialist; *“It’s hospital practice”* – nurse), while others advocated the legal or moral precedence of the ACD *(“It’s morally right to give it precedence”* – nurse; “*It’s a legal document, signed by a Justice of the Peace”* – social worker). Other HCPs claimed precedence should be given to whichever documentation was completed more recently, arguing that each completed document should supersede the last.

### Consequences of ‘routine’ provision

While the widespread awareness and use of 7-SP was deemed beneficial in supporting and scaffolding end-of-life conversations and documentation, the ‘routine’ nature of that documentation was reported to bring with it corresponding complexities. Indeed, some HCPs indicated that the very principles underpinning the 7-SP policy—including patient autonomy and informed consent, as well as clarity of end-of-life decision-making processes and transparency between treating clinicians—were potentially undermined in a system in which 7-SP forms are expected as a matter of course.

For example, patient choice not to engage in end-of-life treatment/care conversations may be undermined by institutional/colleagues’ pressure to complete the form, the first section of which requires a conversation with the patient.

... My biggest problem with the 7-Step Pathway is that the first box in that is ‘are they able to talk to you about it,’ and some can't or won’t because they have unrealistic expectations about what they want. And then for the next, you know, multiple days … all you get is constant hassles from nursing staff saying, “You need to fill in this form.” (oncologist)

This can be problematic in circumstances where there are questions around a patient’s capacity to engage in a rational decision-making process, perhaps owing to the stress of an acute admission or the trajectory of a patient’s disease.

They’re doing 7-Step Pathways … when they’ve got cognitive impairment, delirious, family are having big disagreements. It’s not necessarily what [the patient’s] wishes would have been. (social worker)

### Enhancing ‘clarity’

HCPs agreed that the clarity engendered by writing a 7-SP can “*stop the plan falling apart at midnight in the ED* [Emergency Department] (oncologist),” but some indicated that particular professional skills are required of those expected to produce them while maintaining treating relationships and patient outcomes. An understanding of the role of the 7-SP, and of various professionals’ responsibilities to enact them, was considered essential in this regard:

[Other clinicians] need to understand that they can say no [to treatments for their patients], but they can’t demand. (ICU specialist)

Other ICU specialists highlighted the complexities involved when presented with ICU patients with care plans in which only limitations are clear:

[When we see other HCPs’ plans] they are so incomplete. [The patient’s] not for intervention and not for CPR but if they then still come to us, what more can we do? (ICU specialist)

### Enhancing ‘transparency’

Significantly, HCPs indicated that requirements to ensure ‘transparency’ of clinical care plans at the end-of-life through routine completion of the 7-SP can see this as the only means through which limitations to care, or conservative measures, are perceived to be legitimate.

Even palliative care [staff] sometimes will say, “You don’t have a 7-Step,” and it’s because it’s a complex discussion and … they want us to go there and force that [conversation] down, you know … . So, yes, the problem is that when it’s sort of forced that the [Alert] Form is the only way to have made this possible. (oncologist)

In turn, some HCPs indicated that ACDs and the sensitivities of patients/substitute decision-makers may be overlooked, and clinical judgement around the complexity of end-of-life discussions potentially undermined.

## Discussion

This paper represents an examination of Advance Care Directives and an integrated care pathway (the 7-Step Pathway) as they intersect to guide end-of-life care in an Australian hospital setting. Our analysis suggests that ACDs are seen by HCPs working in acute care as a potentially valuable means of promoting patient autonomy, but as rarely completed and poorly integrated into hospital systems. On the other hand, the process and documentation of the 7-SP was perceived as providing clarity for clinicians regarding their responsibilities for completion, storage, and implementation of an approved care plan, and as a well-understood resource that is fully integrated within hospital systems. Inherent in these accounts was the notion, identified elsewhere, that end of life (EOL) documentation should ideally “strike a balance between patient advocacy and clear medical handover” ([14] p.2). However, participants in this study appeared to emphasise the importance of patient autonomy ‘in principle’, while orienting to a need for relevant, clinically-informed directives ‘in practice’.

While the ‘non-specificity’ of ACDs could be seen as a useful indication of a patient’s enduring preferences, their ‘static’ nature was viewed by acute care HCPs as a limitation to their potential relevance and effectiveness in acute care. To some extent, the 7-SP was held to address the identified deficits of ACDs, in that this documentation was characterised as ‘dynamic,’ ‘context-specific,’ and ‘clinically relevant’. At the same time, HCPs reported concern around the propensity of the 7-SP to ‘reinvent the wheel’ of end-of-life conversations, causing distress for patients and families and compromising therapeutic rapport.

These contrasting perceptions align with findings of another Australian qualitative study, which reported that ACDs were positively viewed by diverse HCPs as “proactive,” while use of clinical guidelines (which, like the 7-SP, featured specific steps to direct clinical decision-making) was negatively viewed as “reactive” ( [22], p. 8). Our analysis reveals some further complexity in this regard in that the perceived benefits of ‘proactive’ ACDs may be undermined if one-off completion simultaneously renders them ‘static’. Conversely, while necessarily ‘reactive’, 7-SP documentation may nevertheless be perceived as providing a more ‘dynamic’ response to healthcare decisions as they become relevant during the course of a patient’s illness.

Our findings suggest that EOL planning would usefully encompass enduring patient values *and* be responsive to an individual’s health trajectory—an approach arguably envisaged in the clinical directive that the 7-SP should “translate the results or outcomes of the 7-Step process, along with any Advance Care Directive (ACD) … into a plan that the clinical care team can put into action” ([12] p.9). Yet, despite their mutual orientation to patient-centred practice, the intersection of ACDs and the 7-SP appears to be fraught within acute care. Issues around ACD accessibility, and confusion around precedence, enactment, signing, and revocation make “translation” into “action” complex. In contrast, and as noted elsewhere [23], the ‘routine integration’ and ‘clarity’ provided by integrated care plans such as the 7-SP appear to support uptake and implementation, but potentially constrain concern about patient autonomy simply to the requirement of ‘transparency’ outlined on the standard form [12].

Importantly, our analysis suggests that the 7-SP may work in practice to (re)produce some of the problems it seeks to solve. As observed by Noble et al. [22], our findings indicate that an institutional emphasis on integrated care plans such as the 7-SP may see nurses exert pressure upon medical practitioners to complete this documentation. This pressure may be applied on the basis of seeking to ensure compliance, or because the incorporation of the 7-SP into routine practice creates the expectation that patient care will be compromised without it–a perspective bolstered by the pervasive promotion of integrated care plans within a ‘quality improvement’ framework [24]. In turn, clinicians may feel compelled to initiate conversations with patients and caregivers that may be unwelcome and, if an ACD is operative, possibly unnecessary. While potentially presenting problems in terms of respecting patients’ choices, this circumstance reflects the tension noted elsewhere [11] between encouraging ‘patient-centred care’ and efforts to standardise care provision in line with best-practice.

Regardless, where the 7-SP is entrenched to the point of routine, something that has been identified as a potentially useful means of scaffolding conversations and as having important symbolic value in legitimising death as an outcome, can potentially be perceived as the only means by which this can be achieved. Where this is the case, the perceived authority of ACDs, or of patients’ choice *not* to participate in end-of-life discussions, may be progressively undermined. In turn, scope for clinicians to bring to bear palliative care principles, technical disease-specific guidance and an orientation to individuals’ needs—factors identified as central to responsive, high-quality end-of-life care provision [24]—may be constrained by a systemic emphasis on standardised forms and procedures.

Finally, the variation within participant perspectives on whether an ACD or 7-SP takes precedence, and when/how they may be combined, suggests a need for further clarity and training on the respective roles of these documents in guiding clinical practice. The use of electronic healthcare records may increase the availability and accessibility of ACDs, and potentially see them better embedded within clinical practice, but further efforts will be needed to address clinician uncertainty or misconceptions where instructions might overlap with 7-SP processes. Others have noted the importance of ongoing training and evaluation in ensuring that mechanisms to promote standards of excellence in EOL care (including integrated care pathways) maintain a focus on patient-centred outcomes, rather than on adherence to standardised procedures [22–25].

### Study limitations

This study recruited from two hospitals within one Australian capital city and thus their views may not represent experiences of other clinicians within other locations. The use of 7-SP and ACD documentation was one part of a larger mixed-methods study focusing on ACD awareness and prevalence, and it is possible that this orientation may have influenced participant responses. A strength of this study was the use of qualitative enquiry allowing for the meaning and application of these documents in practice (independently or in tandem) to be closely scrutinised. Moreover, the large number of participants across different medical, surgical, and allied health provided a comprehensive inclusion of the views of clinicians engaging with 7-SP and/or ACD documents across hospital contexts. We acknowledge, however, that we did not include the views of patients and families, and that future research should do so to realise an ethos of patient-centred care.

## Conclusion

Our analysis suggests that, regardless of their specific form, effective processes for facilitating end-of-life planning in the acute setting would possess certain core components. These include clear and accessible documentation, system integration, open discussions, and an orientation both to patients’ enduring values and context-specific perspectives. At the same time, we observe that quality EOL planning also requires clarity on the respective roles and limitations of different EOL documentation, as well as an informed, well-resourced health workforce who understand the legislative context within which specific documentation is embedded, principles of patient autonomy, and the dynamics associated with collaborative decision-making in end-of-life care.

## Data Availability

The datasets used and/or analysed during the current study are available from the corresponding author on reasonable request.
